# Hypoxaemia and risk of death among children: rethinking oxygen saturation, risk-stratification, and the role of pulse oximetry in primary care

**DOI:** 10.1016/S2214-109X(24)00209-2

**Published:** 2024-06-21

**Authors:** Hamish R Graham, Carina King, Trevor Duke, Salahuddin Ahmed, Abdullah H Baqui, Tim Colbourn, Adegoke G Falade, Helena Hildenwall, Shubhada Hooli, Yewande Kamuntu, Rami Subhi, Eric D McCollum

**Affiliations:** a Melbourne Children's Global Health, Murdoch Children's Research Institute, University of Melbourne, Royal Children's Hospital, Melbourne, VIC, Australia; b Department of Paediatrics, University College Hospital Ibadan, Ibadan, Nigeria; c Department of Global Public Health, Karolinska Institute, Stockholm, Sweden; d Projahnmo Study Group, Johns Hopkins University, Dhaka, Bangladesh; e Department of International Health, Johns Hopkins Bloomberg School of Public Health, Baltimore, MD, USA; f Global Program in Pediatric Respiratory Sciences, Department of Pediatrics, Eudowood Division of Pediatric Respiratory Sciences, Johns Hopkins Bloomberg School of Public Health, Baltimore, MD, USA; g Institute for Global Health, University College London, London, UK; h Department of Paediatrics, University of Ibadan, Ibadan, Nigeria; i Division of Emergency Medicine, Department of Pediatrics, Baylor College of Medicine, Houston, TX, USA; j Essential Medicines, Clinton Health Access Initiative, Kampala, Uganda

## Abstract

Pulse oximeters are essential for assessing blood oxygen levels in emergency departments, operating theatres, and hospital wards. However, although the role of pulse oximeters in detecting hypoxaemia and guiding oxygen therapy is widely recognised, their role in primary care settings is less clear. In this Viewpoint, we argue that pulse oximeters have a crucial role in risk-stratification in both hospital and primary care or outpatient settings. Our reanalysis of hospital and primary care data from diverse low-income and middle-income settings shows elevated risk of death for children with moderate hypoxaemia (ie, peripheral oxygen saturations [SpO_2_] 90–93%) and severe hypoxaemia (ie, SpO_2_ <90%). We suggest that moderate hypoxaemia in the primary care setting should prompt careful clinical re-assessment, consideration of referral, and close follow-up. We provide practical guidance to better support front-line health-care workers to use pulse oximetry, including rethinking traditional binary SpO_2_ thresholds and promoting a more nuanced approach to identification and emergency treatment of the severely ill child.

## Introduction

Since pulse oximeters were invented in the 1970s, they have become indispensable tools for assessing blood oxygen levels in emergency departments, operating theatres, high dependency and intensive care units, and hospital wards. Now recognised as an essential medical device by WHO,^1^ pulse oximeters have been championed by the safe surgery movement,^2^ and gained a spotlight during the COVID-19 pandemic for detection of hypoxaemia (ie, low blood oxygen levels) even among patients at home.^3^ Pulse oximetry is particularly important for paediatric hospital care, with WHO guidelines (ie, the WHO pocketbook of hospital care for children and oxygen therapy in children) recognising hypoxaemia as an indicator of severe illness associated with a range of respiratory, systemic, and post-surgical conditions. These guidelines recommend pulse oximetry for all children with emergency or respiratory signs, recognising the crucial role of pulse oximetry in the identification of hypoxaemia and guidance of oxygen therapy.^4^, ^5^ The guidelines also recommend oxygen therapy for all children with an SpO_2_ of less than 90%. Recent WHO-commissioned reviews have further emphasised hypoxaemia as a major risk factor for death and highlighted the importance of pulse oximetry in risk stratification.^6^

However, there is little clarity on the role of pulse oximetry and interpretation of SpO_2_ outside of hospital settings, particularly when used for triage and risk stratification, rather than only to guide whether or not to give oxygen. The WHO integrated management of childhood illness (IMCI) 2014 chart booklet, the key clinical guideline for use in primary care facilities, contains a single footnoted mention of pulse oximetry: “if pulse oximeter is available, determine oxygen saturation and refer if <90%”.^7^ The updated 2019 IMCI chart booklet for young infants makes no mention of pulse oximetry or hypoxaemia.^8^

In this Viewpoint, we review existing evidence and reanalyse data from patients admitted to secondary-level health facilities and patients receiving care at outpatient primary or secondary care facilities to explore three related questions. First, how should different levels of hypoxaemia in children be interpreted, particularly in outpatient settings? Second, what is the role of pulse oximetry screening for children in the outpatient setting? Third, can a particular SpO_2_ cutoff guide risk-stratification and triage decision-making?

## How should different levels of hypoxaemia be interpreted?

Normal blood oxygen levels exist on a continuum and vary by altitude. At sea level, typical arterial blood oxygen saturation is broadly accepted to be equal to or greater than 94% for all age groups (partial pressure of oxygen ∼75–100 mm Hg).^5^ Data from healthy child and adult populations show that typical arterial blood oxygen saturation in the 5th centile is approximately 92–96% at sea level and approximately 88–92% at 2500 m above sea level.^9^, ^10^ Data from children in hospital with pneumonia show that hypoxaemia on admission to hospital is the strongest predictor of death, with the risk of death varying between studies and settings, but proportional to the severity of hypoxaemia, and elevated even at moderate hypoxaemia of 90–93%.^6^ Although the prevalence of hypoxaemia is decreased in non-pneumonia conditions, such as malaria and meningitis, the presence and severity of hypoxaemia is similarly associated with death, as hypoxaemic respiratory failure is a complication of many critical systemic illnesses.^11^, ^12^, ^13^, ^14^, ^15^

To better compare the risk of death across different levels of hypoxaemia and for different child populations (ie, children aged <12 years), we conducted a secondary analysis of diverse study datasets, which include two inpatient study populations (Nigeria^12^ and Uganda [unpublished]) and four primary care or outpatient care study populations (Nigeria,^16^ Malawi,^17^ Malawi,^18^ and Bangladesh^19^), all in low-altitude settings (table). All studies provided data on SpO_2_ from the time of patient presentation, as well as clinical outcome data assessed at discharge or 14–21 days follow-up (see appendix pp 2–6 for individual study details). Using aggregated data on SpO_2_ on presentation and mortality, we calculated the relative odds of death for different SpO_2_ ranges versus an SpO_2_ of 98–100%.

**Table:** Summary of odds of death and case fatality rates for children included in four primary care or outpatient care studies and two inpatient studies, by initial SpO_2_ level

		**Primary care or outpatient care studies**					**Inpatient studies**			**Total**
		**Nigeria INSPIRING**^16^	Malawi EREMISS^17^	Malawi health worker study^18^	Bangladesh Upazila^19^	Subtotal primary care or outpatient care studies	Nigeria O_2_ project^12^	Uganda O_2_ project (unpublished)	Subtotal inpatient studies	
Population		Children younger than 5 years with clinical pneumonia	Children younger than 12 years referred	Children younger than 5 years with pneumonia	Children aged 3–36 months with difficulty breathing	..	Children aged 1–60 months admitted	Children aged 1–60 months admitted	..	..
Number of patients		2683	782	1112	7083	11 660	12 570	26 046	38 616	49 164
Deaths (case fatality rate)		22 (0·82%)	32 (4·1%)	30 (2·7%)	37 (0·52%)	121 (0·86%)	558 (4·4%)	434 (1·7%)	992 (2·6%)	1083 (2·2%)
Deaths, OR of deaths compared with 98–100% SpO_2_										
	SpO_2_ 98–100%	n=3, 1 (ref)	n=6, 1 (ref)	n=6, 1 (ref)	n=11, 1 (ref)	n=26, 1 (ref)	n=83, 1 (ref)	n=92, 1 (ref)	n=175, 1 (ref)	n=201, 1 (ref)
	SpO_2_ 96–97%	n=0	n=6, 1·65 (0·52–5·18)	n=5, 0·82 (0·25–2·70)	n=5, 0·62 (0·21–1·78)	n=16,0·87 (0·47–1·62)	n=57, 1·33 (0·94–1·87)	n=54, 0·97 (0·69–1·36)	n=111, 1·09 (0·86–1·39)	n=127, 1·03 (0·83–1·29)
	SpO_2_ 94–95%	n=2, 2·52 (0·12–15·14)	n=5, 2·28 (0·68–7·62)	n=5, 2·01 (0·60–6·69)	n=5, 1·39 (0·48–4·00)	n=17, 2·04 (1·10–3·77)	n=38, 1·76 (1·19–2·60)	n=26, 1·18 (0·76–1·83)	n=64, 1·49 (1·12–1·99)	n=81, 1·52 (1·18–1·98)
	SpO_2_ 92–93%	n=2, 6·08 (1·01–36·7)	n=4, 3·68 (1·00–13·47)	n=3, 3·21 (0·78–13·21)	n=5, 3·89 (1·34–11·24)	n=14, 4·42 (2·29–8·51)	n=28, 1·79 (1·16–2·77)	n=28, 2·74 (1·78–4·20)	n=56, 2·41 (1·78–3·27)	n=70, 2·68 (2·03–3·53)
	SpO_2_ 90–91%	n=1, 5·04 (0·52–48·99)	n=0	n=1, 1·42 (0·17–12·07)	n=3, 6·76 (1·86–24·54)	n=5, 3·28 (1·25–8·61)	n=26, 3·11 (1·98–4·89)	n=28, 3·90 (2·54–5·98)	n=54, 3·66 (2·68–4·99)	n=59, 3·77 (2·81–5·07)
	SpO_2_ 88–89%	n=2, 20·41 (3·32–125·65)	n=3, 10·6 (2·41–47·03)	n=1, 2·98 (0·34–26·04)	n=1, 4·14 (0·53–32·51)	n=7, 9·45 (4·03–22·15)	n=24, 6·56 (4·07–10·59)	n=28, 8·44 (5·46–13·05)	n=52, 7·76 (5·63–10·70)	n=59, 8·20 (6·08–11·07)
	SpO_2_ 86–87%	n=1, 15·3 (1·54–152·12)	n=0	n=1, 8·10 (0·86–76·46)	n=0	n=2, 5·07 (1·18–21·77)	n=11, 5·46 (2·82–10·56)	n=16, 7·99 (4·62–13·81)	n=27, 6·87 (4·51–10·45)	n=29, 7·00 (4·68–10·47)
	SpO_2_ ≤85%	n=10, 41·9 (11·34–154·81)	n=6, 8·28 (2·54–27·03)	n=6, 8·95 (2·75–29·12)	n=3, 25·2 (6·72–94·51)	n=25, 23·2 (13·18–40·95)	n=174, 15·6 (11·83–20·56)	n=133, 17·1 (12·98–22·49)	n=307, 4·06 (3·37–4·90)	n=332, 19·7 (16·39–23·60)
	SpO_2_ data missing	n=1, 3·71 (0·38–36·07)*	n=2, 12·4 (2·16–71·28)*	n=2, 1·17 (0·23–5·88)	n=4, 8·34 (2·62–26·53)*	n=9, 4·90 (2·28–10·53)	n=117, 1·91 (1·44–2·54)	n=29, 1·01 (0·66–1·53)	n=146, 1·86 (1·49–2·32)	n=1155, 2·27 (1·84–2·80)

Data are presented as n, OR (95% CI), unless otherwise indicated. EREMISS=emergency paediatric treatment and referral in Malawi in frontline health-care settings. INSPIRING=INtegrated Sustainable childhood Pneumonia and Infectious disease Reduction in Nigeria. OR=odds ratio.

* Missing data primarily due to attempted but failed oximeter reading (other missing included not attempted).

Reviewing the individual study data showed that risk of death varied substantially between the different settings and study populations (table). As expected, higher case fatality rates were observed among the inpatient and referred patient groups (case fatality rate 2–4%) compared with the general outpatient populations (case fatality rate <1%). However, across all datasets there was a consistent trend towards higher odds of death at SpO_2_ values lower than 96%. Odds of death were also high among individuals for whom pulse oximetry was attempted, but an SpO_2_ measurement was not obtained, confirming data from previous studies.^20^

Combining these data and plotting a fitted exponential curve of the odds of deaths clarified this trend. The odds of death doubled at SpO_2_ of 94–95% compared with 98–100%, almost tripled at 92–93%, quadrupled at 90–91%, and increased 6-fold at 88–89% (figure). This pattern is almost identical when restricting to the four primary-care or outpatient-care studies or the two in-hospital studies and remains robust in a sensitivity analysis that equally weights all studies (appendix pp 8, 9). These data show SpO_2_ as a potent tool for risk stratification, with odds of death increasing with decreasing SpO_2_ similarly across diverse inpatient and outpatient populations. These data especially illustrate the considerable increased odds of death among those individuals with moderate hypoxaemia in the SpO_2_ 90–93% range.

**Figure:**
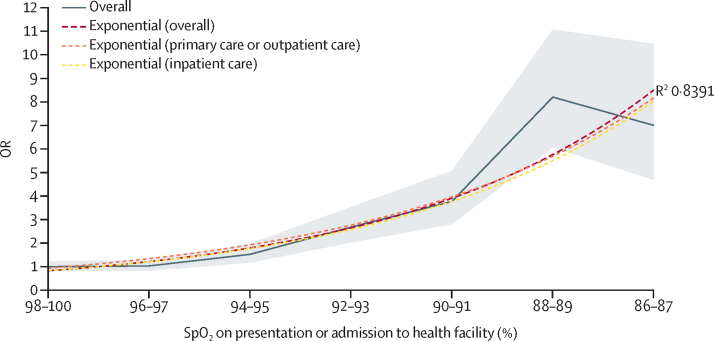
Death for children with different SpO_2_ values OR of death presented for children with different SpO_2_ values compared with children with SpO_2_ 98–100%. Overall OR is presented in grey with 95% CI shaded in light grey. Fitted exponential trend lines show ORs overall and by facility type (ie, individuals in primary care and outpatient care *vs* individuals admitted to health facility). An extended graph, including SpO_2_ less than 86%, is available in the appendix (pp 8, 9). OR=odds ratio. SpO_2_=peripheral oxygen saturation.

## What is the role of pulse oximetry in pre-hospital settings?

Recognising the considerable increase in odds of death for children with moderate hypoxaemia (SpO_2_ 90–93%) has important implications for risk stratification in primary care facilities. In addition to guiding oxygen therapy, clinicians increasingly recognise that pulse oximetry is a valuable risk assessment tool that can assist in: identifying children who should be reassessed for any missed clinical danger signs; guiding referrals from pre-hospital settings; informing resource allocation for patients attending a facility; and identifying patients who might benefit from closer follow-up after returning to the community.^21^

Currently, WHO guidelines use a range of clinical danger or emergency signs to identify severely ill children who require referral and inpatient care.^7^ Some clinical signs, such as coma or signs of shock, are highly predictive of poor clinical outcomes, but are relatively uncommon (ie, high specificity, low sensitivity). Other clinical signs, such as inability to feed, are less strongly associated with death, but are relatively common in unwell young children (ie, high sensitivity, low specificity). In addition, many of these clinical signs are subjective and even the best collection of clinical signs misses children who die.^22^, ^23^

Previous studies have found that the use of pulse oximetry adds greatly to the sensitivity of WHO clinical assessment of severe childhood illness. A study in Bangladesh found that, among approximately 4000 children aged 3–11 months with suspected pneumonia, WHO IMCI guidelines implemented without oximetry missed 90 (88%) of 102 children with severe hypoxaemia (ie, SpO_2_ <90%), including all four severely hypoxaemic children who subsequently died.^19^ Data from approximately 13 000 children attending rural health centres in Malawi found that one-third of children with severe hypoxaemia (SpO_2_ <90%, n=652) would not have been identified for referral without oximetry.^24^ However, hypoxaemia also has good specificity, as it is rarely detected in healthy populations,^10^ and its prevalence in community settings and general primary care populations is relatively low.^19^, ^25^, ^26^

These data support calls for SpO_2_ to be regarded as a vital sign, with pulse oximetry checks done routinely on all acutely unwell children alongside temperature, heart rate, respiratory rate, and weight.^27^ However, although routinely conducted pulse oximetry is increasingly recognised as a standard of care in hospitals, there have been questions about feasibility, effectiveness, and cost in extending pulse oximetry services to primary care and outpatient facilities. To date, there have been few examples of successful implementation of pulse oximetry in primary care settings^28^, ^29^ (although large-scale roll-outs are underway in an increasing number of countries^30^) and core barriers and implementation questions need to be resolved. First, staff capacity and workload challenges need to be addressed; paediatric oximetry measurements often take 1–2 min to achieve and potentially shift time away from other activities in a busy clinical context (even in primary care settings). Second, pulse oximeters must be appropriate for children and front-line settings, including having available appropriately sized probes for small children, which are accurate across skin tones and with motion, low-cost, robust, and with good battery life.^31^, ^32^ Third, the introduction of pulse oximetry into routine care requires more than simply providing oximeters and training staff; roll-out must fit local needs, context, equipment management, and care processes.^33^ Fourth, it is essential to determine the right target population: if it is not feasible to identify all acutely unwell children presenting to health facilities, assessing children with fever, respiratory, or any emergency signs might prove more feasible. Fifth, the broad health system impacts of changing assessment and referral or follow-up guidelines (eg, increased numbers of referrals or follow-up visits) must be anticipated and improvements in referrals and hospital quality of care must be supported, so that acutely unwell children will get the care that they need.

## Can a particular SpO_2_ cutoff guide risk-stratification and triage decision-making?

As child health policy makers and programme managers define the role of pulse oximetry in primary care settings, a common question is which SpO_2_ cutoff to choose and for what decision. In the absence of clear guidance from WHO, some countries (eg, Niger and Guinea) have adopted the traditional SpO_2_ cutoff of less than 90% to trigger a referral to high-level health facilities, whereas others have opted for higher thresholds of less than 93% in Mali or less than 95% in Burkina Faso.^34^

From a clinical perspective, SpO_2_ is a dynamic clinical parameter that must be interpreted in the context of each patient and their care setting. For example, a young child with bronchiolitis and an SpO_2_ of 92%, but no other comorbidities or risk factors, is at much lower risk than a child with the same SpO_2_ alongside other evidence of clinical instability, such as seizures, reduced conscious state, or shock. Furthermore, the appropriate management of these children will depend on context, for example, different management will be available to a patient presenting to a remote health post, as compared with a higher capacity primary care facility offering day care admissions under medical oversight, or a major hospital with dedicated paediatric staff.

The traditional hypoxaemia cutoff (ie, SpO_2_ <90%) was primarily intended to define who should receive oxygen therapy at low altitude.^4^ This cutoff threshold comes with a caveat of targeting higher SpO_2_ for emergency resuscitation,^35^ and particular conditions (eg, severe anaemia and heart failure), which are highly prevalent among children who are living in settings where malnutrition, micronutrient deficiencies, and malaria are common.^36^ However, when pulse oximetry is also used for risk-stratification, the goal is to identify severely ill children who need additional attention, regardless of their need for oxygen or any other particular therapy.

Our data showed similar odds of death for children with an SpO_2_ of 96–97% compared with 98–100%, and marginally higher odds of death for children with an SpO_2_ of 94–95%. However, moderate hypoxaemia in the range of SpO_2_ 90–93% carried an increased odds of death, which is similar to findings from other studies that have reported general danger signs, severe malnutrition, or cyanosis conditions, which should almost always result in referral to a high-level facility.^6^ Although severe hypoxaemia (SpO_2_ <90%) should be a clear indication for referral and admission to a high-level facility, we suggest that moderate hypoxaemia (SpO_2_ 90–93%) and so-called failed readings should also be considered as clear cause for health-care worker concern and action. What this looks like will depend on the local context and capabilities of the health facility, with some primary care facilities able to offer day-care or short admissions with experienced oversight, giving health-care workers time to observe the child to see whether they are clinically stable, whereas other facilities without this capacity rely more on referral pathways to higher-level facilities.

## Maryam's story

Here we present a hypothetical case study to contextualise some of the issues addressed in this Viewpoint.

Tuesday morning at Goro clinic, a small primary-care facility providing basic outpatient services to the surrounding rural population, a 2-h drive from the closest hospital. The solo clinical officer, Maryam, surveys the busy waiting room as she calls the next patient in. A mother comes forward with a young child in her arms. The child looks at Maryam warily and holds tightly to her mother. Maryam smiles. “Oh my little one, you don't look well. What is wrong?” The story tumbles out: the child is 18 months old, was previously well but now has had persistent fevers for 3 days, is still drinking but not interested in food, took malaria medication from the drugstore 2 days ago, and still not getting better. Maryam approaches the child gently, noticing that her breathing is fast and a little laboured. She counts the breathing rate (50 breaths per min), listens to her breathing (no wheeze), and then places the pulse oximeter probe on her toe. The child pulls away but is reassured by her mother. The oximeter screen lights up showing a fast but regular heartbeat (140 beats per min) and an SpO_2_ of 91%.

### What should Maryam do?

Maryam can see that the young child is presenting with fever, fast breathing, mild chest indrawing, no WHO danger signs, but evidence of moderate hypoxaemia (SpO_2_ of 91%). Following current WHO IMCI advice, Maryam would be correct in giving oral antibiotics for home treatment of pneumonia and arranging outpatient review. However, we suggest that this child should be recognised as high-risk, prompting Maryam to carefully repeat her clinical assessment in case she has missed any important signs. Given that she is the most senior medical officer at a small, rural, primary care clinic, Maryam does not have the ability to admit or observe this child and should strongly consider referral to the district hospital, a 2-h drive away. Finally, Maryam should arrange follow-up to ensure that the child receives appropriate care and makes a full recovery. To make these decisions and support the family to get appropriate care, Maryam needs clear, evidence-based guidance in her clinical protocols and reasonable confidence in the accessibility and quality of care that she is recommending (both locally and at the referral centre). To accept Maryam's recommendation, the family need confidence that the recommendation is authoritative and reasonable, and that they will be treated well by staff at the larger facility and locally when they return for review.

## Implications for policy and practice

Although there are outstanding questions regarding the effectiveness, acceptability, cost, and health systems implications of increasing the use of pulse oximetry in primary care and outpatient settings and broadening the criteria for referral to hospital, there are some findings that can be acted on now.

First, pulse oximetry has important risk assessment value to health-care workers that should be more fully used. Hypoxaemia should be recognised as an important danger sign in both hospital and pre-hospital clinical guidelines, with severe hypoxaemia (SpO_2_ <90%), moderate hypoxaemia (SpO_2_ 90–93%), and failed readings all associated with substantially higher risk of death than normoxaemia (SpO_2_ ≥94%).

Second, detection of moderate hypoxaemia (SpO_2_ 90–93%) or a failed reading in a child presenting to a primary care facility should prompt three actions: careful clinical reassessment; consideration of referral to a higher-level facility; and close follow-up within and after leaving the facility.

Third, governments and other implementing agencies seeking to expand the use of pulse oximetry in primary care and outpatient settings should understand that it is one tool that exists alongside other clinical tools and processes, not a standalone technological intervention. For pulse oximetry to be adopted and have impact, health-care workers will need ongoing support to integrate it into their workflows for the benefit of patients and current workforce shortages will need to be addressed.

Fourth, reducing preventable mortality from pneumonia and other severe acute illnesses requires better identification of severely ill children in the community and pathways for them to receive quality, context-appropriate care. Although pulse oximetry might be a valuable adjunctive tool for the identification of severely ill children presenting to small facilities, we also need greater efforts to improve referral pathways and quality of care in general hospitals, and capacity to follow up children in the community.

Fifth, pulse oximeters are an essential medical device and should be more accessible globally, particularly in poor and rural areas. Increasing oximeter accessibility requires greater availability of oximeters that are affordable, robust, easy to use, and appropriate for the target population (including infants and children and people with deeply pigmented skin tones).

### Contributors

## Declaration of interests

All authors have received institutional fellowship or grant funding related to pulse oximetry and oxygen research, including funding to conduct the original studies reanalysed in this Viewpoint: Save the Children UK (AGF, TC, CK, and HRG); Bill & Melinda Gates Foundation (AGF, TD, HRG, YK, EDM, AHB, SA, and TC); ELMA Philantrophies (YK); Swedish Research Council (HH, CK, and EDM); Einhorn Family Foundation (HH); Wellcome Trust (TC, CK, and EDM); National Institutes of Health (EDM and SH); WHO (TC, EDM, AHB, SA, and TC); United States Agency for International Development (EDM); US Centers for Disease Control and Prevention (EDM); Thrasher Research Fund (EDM); Moderna (EDM); National Health and Medical Research Council (HRG); and Royal Children's Hospital Foundation (HRG). YK is employed by Clinton Health Access Initiative, which contributes to global pulse oximetry and oxygen strategy. TC has received consulting fees from the UN and chairs a steering committee for an adolescent mental health trial in Nepal. HRG and EDM have served as advisers to the Tools for Integrated Management of Childhood Illness–Améliorer l'Identification des détresses Respiratoires chez l'Enfant (AIRE) pulse oximetry trial. EDM co-chairs the Union Child Pneumonia Working Group; HRG and CK co-chair the *Lancet Global Health* Commission on Medical Oxygen. AGF and HRG are board members for Oxygen for Life Initiative, a non-profit research and implementation group for pulse oximetry and oxygen. HRG, CK, and EDM have served as advisers to Lifebox Foundation, a non-governmental organisation that aims to increases access to pulse oximetry.
