# Supplementary material for: Hypoxaemia and risk of death among children: rethinking oxygen saturation, risk-stratification, and the role of pulse oximetry in primary care
Source: Lancet Glob Health. 2024 Jun 21;12(8):e1359–64. doi: 10.1016/S2214-109X(24)00209-2 (PMC11254785; doi:10.1016/S2214-109X(24)00209-2)

THE LANCET
Global Health

## Supplementary appendix

This appendix formed part of the original submission and has been peer reviewed. We post it as supplied by the authors.

Supplement to: Graham HR, King C, Duke T, et al. Hypoxaemia and risk of death among children: rethinking oxygen saturation, risk-stratification, and the role of pulse oximetry in primary care. *Lancet Glob Health* 2024; published online June 21. https://doi.org/10.1016/S2214-109X(24)00209-2.

# Contents

Text S1 Additional information about the included studies ........ 2
Bangladesh Upazila study ........ 2
Malawi EREMISS study ........ 3
Nigeria INSPIRING Lagos study ........ 4
Malawi CHW/HC study ........ 5
Nigeria Oxygen Implementation project ........ 6
Uganda CHAI oxygen study ........ 7
Figure S1 Sensitivity analyses ........ 8
Relative odds of death for children with varying peripheral blood oxygen saturation ($SpO_2$) compared to children with $SpO_2$ 98-100% with fitted exponential trend lines, overall and by facility type. ........ 8
A) Each study given equal weighting ........ 8
B) Analysis extended to ≤85% group ........ 8
C) Analysis showing Primary care / Outpatient total and 95% confidence interval ........ 9

# Text S1 Additional information about the included studies

## Bangladesh Upazila study

- **Data collection dates:** September 2015 to August 2017.
- **Setting:** Zakiganj, Kanaighat, and Beanibazar subdistricts, Sylhet District, Sylhet Division, Bangladesh – all government outpatient clinics for under-five year old children at Upazila Health Complexes
- **Population:** Children aged 3 to <36 months presenting to outpatient care with difficult breathing (or suspected pneumonia or lower respiratory infection).
    - 7,083 children 3 to <36 months old with completed follow-up
    - 37 died (0.5% case fatality rate)
- **$SpO_2$ assessment:** Trained study physician did an $SpO_2$ assessment breathing in room air after the child on the same day as enrollment into the study. Used Masimo Rad5 pulse oximeter with y-sensor wrap and were training to wait for a quality signal (defined as waiting 15 seconds, then assessing plethysmography bar and required to be consistent and green in color, stable $SpO_2$ value for 3 seconds). Monthly to every other month supervision from a pediatric pulmonologist, more frequent supervision by senior study physician.
- **Mortality assessment:** Participants followed prospectively utilizing core community surveillance system, outcome at 2-weeks after enrolment was extracted from the surveillance system as the primary endpoint. Deaths were verified by verbal autopsy.
- **Oxygen assessment:** Inpatient data collected from referral hospital.
- **Main publication:** https://www.thelancet.com/journals/lanres/article/PIIS2213-2600(23)00098-X/fulltext

| $SpO_2$ value | Mortality (n/N, %) | | |
|---|---|---|---|
| | Overall | Received oxygen | Did not receive oxygen |
| 100% | 1/467 (0.2%) | 0/4 (0%) | 1/462 (0.2%) |
| 99% | 2/1,236 (0.2%) | 0/4 (0%) | 2/1,232 (0.2%) |
| 98% | 8/1,357 (0.6%) | 0/11 (0%) | 8/1,338 (0.6%) |
| 97% | 3/1,150 (0.3%) | 0/26 (0%) | 3/1,121 (0.3%) |
| 96% | 2/1,105 (0.2%) | 0/24 (0%) | 2/1,081 (0.2%) |
| 95% | 4/629 (0.6%) | 1/27 (3.7%) | 3/599 (0.5%) |
| 94% | 1/375 (0.3%) | 1/19 (5.3%) | 0/356 (0%) |
| 93% | 4/210 (1.9%) | 2/14 (14.3%) | 2/194 (1.0%) |
| 92% | 1/152 (0.7%) | 1/16 (6.2%) | 0/136 (0%) |
| 91% | 2/71 (2.8%) | 0/8 (0%) | 2/63 (3.2%) |
| 90% | 1/55 (1.8%) | 0/4 (0%) | 1/51 (2.0%) |
| 89% | 0/25 (0%) | 0/6 (0%) | 0/19 (0%) |
| 88% | 1/43 (2.3%) | 0/12 (0%) | 1/30 (3.2%) |
| 87% | 0/20 (0%) | 0/3 (0%) | 0/17 (0%) |
| 86% | 0/15 (0%) | 0/2 (0%) | 0/13 (0%) |
| ≤85% | 3/36 (8.3%) | 2/8 (25.0%) | 1/28 (3.6%) |
| Failed $SpO_2$ | 4/137 (2.9%) | 3/30 (10.0%) | 1/107 (0.9%) |
| Overall | 37/7,083 (0.5%) | 10/218 (4.6%) | 27/6,838 (0.4%) |

Malawi EREMISS (Emergency paediatric treatment and referral in Malawi in frontline healthcare settings) study

- **Data collection dates:** July 2019 to April 2020.
- **Setting:** Mchinji district, central region, Malawi – all government primary healthcare facilities (PHCs).
- **Population:** Children aged 0 months - 12 years who were recommended for referral from PHCs to hospital by HCWs during routine care. PHCs implement IMCI, but meeting IMCI criteria was not a requirement, any reason for referral was accepted.
    - 826 children
    - 784 children with completed follow-up
    - 32 died (4.1% case fatality rate)
- **SpO2 assessment:** Trained non-clinical study staff did an SpO2 assessment after the child had been recommended for referral, before they left the PHC. Used Lifebox (Acare) oximeter with both paediatric and adult clip probes, and were trained to wait for a stable waveform. Bi-weekly supervision from a clinical officer.
- **Mortality assessment:** 2-week telephone follow-up, with in-person community visits for those without access to a mobile phone. We also monitored the inpatient wards at the 3 functional CHAM hospitals and district hospital for inpatient mortality. Deaths were meant to be verified by verbal autopsy, but these were largely incomplete when COVID-19 meant we stopped the study early.
- **Oxygen assessment:** Daily monitoring of recruited participants who we registered as inpatients, and telephone questions around treatment and admission at the 2-week follow-up.
- **Main publication:** https://www.ncbi.nlm.nih.gov/pmc/articles/PMC9047421/pdf/BLT.21.287265.pdf

| SpO2 value | Mortality (n/N, %) | | |
|---|---|---|---|
| | Overall | Received oxygen | Did not receive oxygen |
| 100% | 1/21 (4.8%) | 1/1 (100%) | 0/20 (0%) |
| 99% | 3/93 (3.2%) | 0/2 (0%) | 3/91 (3.3%) |
| 98% | 2/190 (1.1%) | 0/6 (0%) | 2/184 (1.1%) |
| 97% | 5/112 (4.5%) | 2/3 (66.7%) | 3/109 (2.8%) |
| 96% | 1/75 (1.3%) | 0/3 (0%) | 1/72 (1.4%) |
| 95% | 5/68 (7.4%) | 2/5 (40.0%) | 3/60 (2.8%) |
| 94% | 0/46 (0%) | 0/2 (0%) | 0/44 (0%) |
| 93% | 2/27 (7.4%) | 0/5 (0%) | 2/22 (9.1%) |
| 92% | 2/31 (6.5%) | 2/5 (40.0%) | 0/28 (0%) |
| 91% | 0/18 (0%) | 0/4 (0%) | 0/14 (0%) |
| 90% | 0/26 (0%) | 0/6 (0%) | 0/20 (0%) |
| 89% | 3/12 (25.0%) | 1/2 (50.0%) | 2/10 (20.0%) |
| 88% | 0/5 (0%) | 0/2 (0%) | 0/3 (0%) |
| 87% | 0/5 (0%) | 0/1 (0%) | 0/4 (0%) |
| 86% | 0/1 (0%) | 0/1 (0%) | - |
| ≤85% | 6/42 (14.3%) | 4/18 (22.2%) | 2/24 (8.3%) |
| Failed $SpO_2$ | 2/10 (20.0%) | 1/2 (50.0%) | 1/8 (12.5%) |
| Overall | 32/784 (4.1%) | 13/68 (19.1%) | 19/716 (2.7%) |

Nigeria INSPIRING (Integrated Sustainable childhood Pneumonia and Infectious disease Reduction in Nigeria) Lagos study

- **Data collection timing:** July 2020 to October 2022.
- **Setting:** Ikorodu LGA, Lagos State, Nigeria. Peri-urban slum area with high density of healthcare facilities (public and private), and 2 secondary facilities.
- **Population:** Children age 0-59 months, diagnosed with WHO IMCI pneumonia by clinical study staff, presenting to either PHC facilities or outpatient departments of secondary facilities.
    - 26,605 children screened
    - 2,683 eligible and completed follow-up
    - 22 died (0.8% case fatality rate)
- **SpO2 assessment:** Study staff, trained in pulse oximetry by Oxygen for Life, conducted an SpO2 assessment before the child had been routinely assessed by HCWs. Equipped with either the Masimo RadG with a paediatric clip probe or the Lifebox (Acare) with both a paediatric and adult clip probe. Assignment was randomised by clinic.
- **Mortality assessment:** 2-week telephone follow-up interview.
- **Oxygen assessment:** Recorded either by study staff, based on the child's clinical notes or observation of oxygen receipt while they were in study clinics, or reported by caregivers in the 2-week telephone follow-up.
- **Main publication:** https://pubmed.ncbi.nlm.nih.gov/35501079/

| SpO2 value | Mortality (n/N, %) | | | |
|---|---|---|---|---|
| | Overall | Received oxygen | Did not receive oxygen | Oxygen status unknown |
| 100% | 1/52 (1.9%) | | | |
| 99% | 0/398 (0%) | | | |
| 98% | 2/747 (0.3%) | | | |
| 97% | 0/365 (0%) | | | |
| 96% | 0/309 (0%) | | | |
| 95% | 2/188 (1.1%) | | | |
| 94% | 0/130 (0%) | | | |
| 93% | 2/88 (2.3%) | | | |
| 92% | 0/45 (0%) | | | |
| 91% | 1/35 (2.9%) | | | |
| 90% | 0/45 (0%) | | | |
| 89% | 1/20 (5.0%) | | | |
| 88% | 1/21 (4.8%) | | | |
| 87% | 1/12 (8.3%) | | | |
| 86% | 0/15 (0%) | | | |
| ≤85% | 10/105 (9.5%) | | | |
| Failed SpO2 | 1/108 (0.9%) | | | |

## Malawi CHW/HC study

- Data collection dates: January 2012 to June 2014.
- **Setting**: Lilongwe (rural – Kabadula health area) and Mchinji districts, central region, Malawi – outpatients at 38 government community health worker (CHW) clinics and 18 government primary health centres (HC).
- **Population**: Children aged 0-59 months old diagnosed with pneumonia.
    - 7,358 CHW and 6,546 HC pneumonia episodes
    - 417 CHW and 695 HC pneumonia episodes were linked to 30-day mortality outcomes
    - 16 (3.8%) CHW and 13 (1.9%) HC patients died
- **SpO2 assessment**: Trained (1-day) and mentored (monthly) health workers measured SpO2 (during routine care, so only one measurement taken). using Lifebox (Acare) pulse oximeters with universal adult clip probe applied to the child's big toe if less than 2 years of age or below 10 kg; or, for older/heavier children an appropriately sized finger. Providers were trained to record measurements that demonstrated consistent plethysmography waveforms along with a stable, non-drifting SpO2 and age-appropriate pulse rate.
- **Mortality assessment**: Matching of CHW and HC datasets to mortality surveillance data from a separate study
- **Oxygen assessment**: Patients with low oxygen saturation were referred though whether oxygen therapy was given was not recorded in the data made available for this study. It is likely that very few were given oxygen therapy given data collection in 2012-2014 when oxygen was not available in many facilities.
- **Main publication**: https://journals.plos.org/plosmedicine/article?id=10.1371/journal.pmed.1003300

| SpO2 value | Mortality (n/N, %) | | |
|---|---|---|---|
| | CHW | HC | Overall |
| 100% | 0/9 (0%) | 0/47 (0%) | 0/56 (0%) |
| 99% | 3/42 (7.1%) | 0/80 (0%) | 3/122 (2.5%) |
| 98% | 3/74 (4.1%) | 0/94 (0%) | 3/168 (17.9%) |
| 97% | 3/103 (2.9%) | 0/70 (0%) | 3/173 (1.7%) |
| 96% | 2/85 (2.4%) | 0/94 (0%) | 2/179 (1.1%) |
| 95% | 1/29 (3.5%) | 2/62 (3.2%) | 3/91 (3.3%) |
| 94% | 0/9 (0%) | 2/46 (4.4%) | 2/55 (3.6%) |
| 93% | 1/5 (20%) | 1/22 (4.6%) | 2/27 (7.4%) |
| 92% | 0/2 (0%) | 1/27 (3.7%) | 1/29 (3.4%) |
| 91% | 0/4 (0%) | 0/17 (0%) | 0/21 (0%) |
| 90% | 1/1 (100%) | 0/19 (0%) | 1/20 (5%) |
| 89% | 0/3 (0%) | 1/10 (0%) | 1/13 (7.7%) |
| 88% | 0/1 (0%) | 0/6 (0%) | 0/7 (0%) |
| 87% | 0 | 1/5 (20%) | 1/5 (20%) |
| 86% | 0 | 0/3 (0%) | 0/3 (0%) |
| ≤85% | 1/3 (33%) | 5/41 (12.2%) | 6/44 (13.6%) |
| Failed $SpO_2$ | 1/47 (2.1%) | 1/52 (1.9%) | 2/99 (2.0%) |
| Overall | 16/417 (3.8%) | 13/695 (1.9%) | 29/1112 (2.6%) |

Nigeria Oxygen Implementation project

- **Setting:** Secondary level health facilities in four states of south-western Nigeria, including government and private not-for-profit facilities.
- **Population:** Children aged 1-59 months, admitted to facilities, for any condition. Dataset restricted to the post-intervention period when pulse oximetry was routinely practiced (>75% coverage).
    - 12570 children included
    - 558 died (4.4% case fatality rate)
- **SpO2 assessment:** Frontline healthcare workers conducted pulse oximetry as part of their routine clinical assessment for pneumonia, using Lifebox (Acare) oximeters with a child clip probe. HCWs were trained in pulse oximetry by study staff and then had ~4-monthly support visits.
- **Mortality assessment:** In-hospital mortality as recorded in case notes. Collated by trained clinical data collectors.
- **Oxygen assessment:** As recorded in case notes. Collated by trained clinical data collectors.
- **Main publication:** https://pubmed.ncbi.nlm.nih.gov/31832620/

| | Mortality (n/N, %) | | | | | | | | | | | |
|---|---|---|---|---|---|---|---|---|---|---|---|---|
| SpO2 value | OVERALL | | | Received oxygen | | | Did not receive oxygen | | | Oxygen status unknown | | |
| | Died | Tot | % | Died | Tot | % | Died | Tot | % | | | |
| 100% | 10 | 458 | 2.2% | 8 | 26 | 30.8% | 2 | 432 | 0.5% | | | |
| 99% | 22 | 1158 | 1.9% | 17 | 86 | 19.8% | 5 | 1072 | 0.5% | | | |
| 98% | 51 | 2462 | 2.1% | 35 | 148 | 23.6% | 16 | 2314 | 0.7% | | | |
| 97% | 29 | 1048 | 2.8% | 22 | 77 | 28.6% | 7 | 971 | 0.7% | | | |
| 96% | 28 | 1075 | 2.6% | 20 | 81 | 24.7% | 8 | 994 | 0.8% | | | |
| 95% | 14 | 584 | 2.4% | 11 | 65 | 16.9% | 3 | 519 | 0.6% | | | |
| 94% | 24 | 494 | 4.9% | 17 | 58 | 29.3% | 7 | 436 | 1.6% | | | |
| 93% | 13 | 314 | 4.1% | 7 | 60 | 11.7% | 3 | 519 | 0.6% | | | |
| 92% | 15 | 467 | 3.2% | 11 | 58 | 19.0% | 7 | 436 | 1.6% | | | |
| 91% | 8 | 165 | 4.8% | 8 | 36 | 22.2% | 0 | 129 | 0.0% | | | |
| 90% | 18 | 263 | 6.8% | 11 | 51 | 21.6% | 7 | 212 | 3.3% | | | |
| 89% | 9 | 69 | 13.0% | 9 | 50 | 18.0% | 0 | 19 | 0.0% | | | |
| 88% | 15 | 131 | 11.5% | 15 | 116 | 12.9% | 0 | 15 | 0.0% | | | |
| 87% | 3 | 48 | 6.3% | 3 | 37 | 8.1% | 0 | 11 | 0.0% | | | |
| 86% | 8 | 60 | 13.3% | 7 | 44 | 15.9% | 1 | 16 | 6.3% | | | |
| ≤85% | 174 | 711 | 24.5% | 168 | 608 | 27.6% | 6 | 103 | 5.8% | | | |
| Missing SpO2 (not necessarily attempted) | 117 | 3063 | 3.8% | 40 | 155 | 25.8% | 38 | 2719 | 1.4% | | | |

## Uganda CHAI oxygen study

- **Setting:** Jinja and Mubende regions, Uganda. Included 2 RRHs, 5 General Hospitals, 24 HCIVs (total 31 facilities). The study was conducted between June 2020 and June 2022 at admitting health facilities with in-patient wards in Busoga and North Buganda regions of Uganda, representing the catchment areas of Mubende and Jinja regional referral hospitals (1100-1400 meters altitude).
- **Population:** Children aged 1 month to 14 years (excluded neonates), admitted to facility. Restricted to the post-intervention period during which 87% of patients had SpO2 documented on admission.
  - 38,936 children admitted
  - 37,483 (96%) with mortality data
  - 584 died (1.6% case fatality rate)
- **SpO2 assessment:** Recorded by study staff based on child's case notes. Conducted by clinical staff, trained in pulse oximetry through MOH/CHAI mentoring program, conducted an SpO2 assessment on admission. Equipped with either handheld oximeter with a paediatric clip probe.
- **Mortality assessment:** In-hospital mortality as recorded in case notes. Missing data on 1453 (3.7%).
- **Oxygen assessment:** Recorded by study staff based on the child's case notes.
- **Main publication:** not yet published**.**

| | Mortality (n/N, %) | | | | | | | | | | | |
|---|---|---|---|---|---|---|---|---|---|---|---|---|
| SpO2 value | OVERALL | | | Received oxygen | | | Did not receive oxygen | | | Oxygen status unknown | | |
| | Died | Tot | % | | | | | | | | | |
| 100% | 13 | 2432 | 0.5% | | | | | | | | | |
| 99% | 42 | 4912 | 1.0% | | | | | | | | | |
| 98% | 71 | 8036 | 0.9% | | | | | | | | | |
| 97% | 30 | 4176 | 0.9% | | | | | | | | | |
| 96% | 41 | 4771 | 0.8% | | | | | | | | | |
| 95% | 21 | 2030 | 0.8% | | | | | | | | | |
| 94% | 24 | 1434 | 1.4% | | | | | | | | | |
| 93% | 9 | 771 | 1.4% | | | | | | | | | |
| 92% | 26 | 793 | 3.3% | | | | | | | | | |
| 91% | 13 | 330 | 5.3% | | | | | | | | | |
| 90% | 20 | 766 | 2.5% | | | | | | | | | |
| 89% | 19 | 249 | 8.6% | | | | | | | | | |
| 88% | 17 | 247 | 5.5% | | | | | | | | | |
| 87% | 9 | 134 | 6.2% | | | | | | | | | |
| 86% | 12 | 159 | 7.1% | | | | | | | | | |
| ≤85% | 167 | 1236 | 13.2% | | | | | | | | | |
| Missing SpO2 (not necessarily attempted) | 42 | 4881 | 0.9% | | | | | | | | | |

Figure S1 Sensitivity analyses

Relative odds of death for children with varying peripheral blood oxygen saturation ($SpO_2$) compared to children with $SpO_2$ 98-100% with fitted exponential trend lines, overall and by facility type.

A) Re-analysis by combining the individual study estimates without weighting for study size (the original analysis pools the data, with larger studies thereby contributing more data)

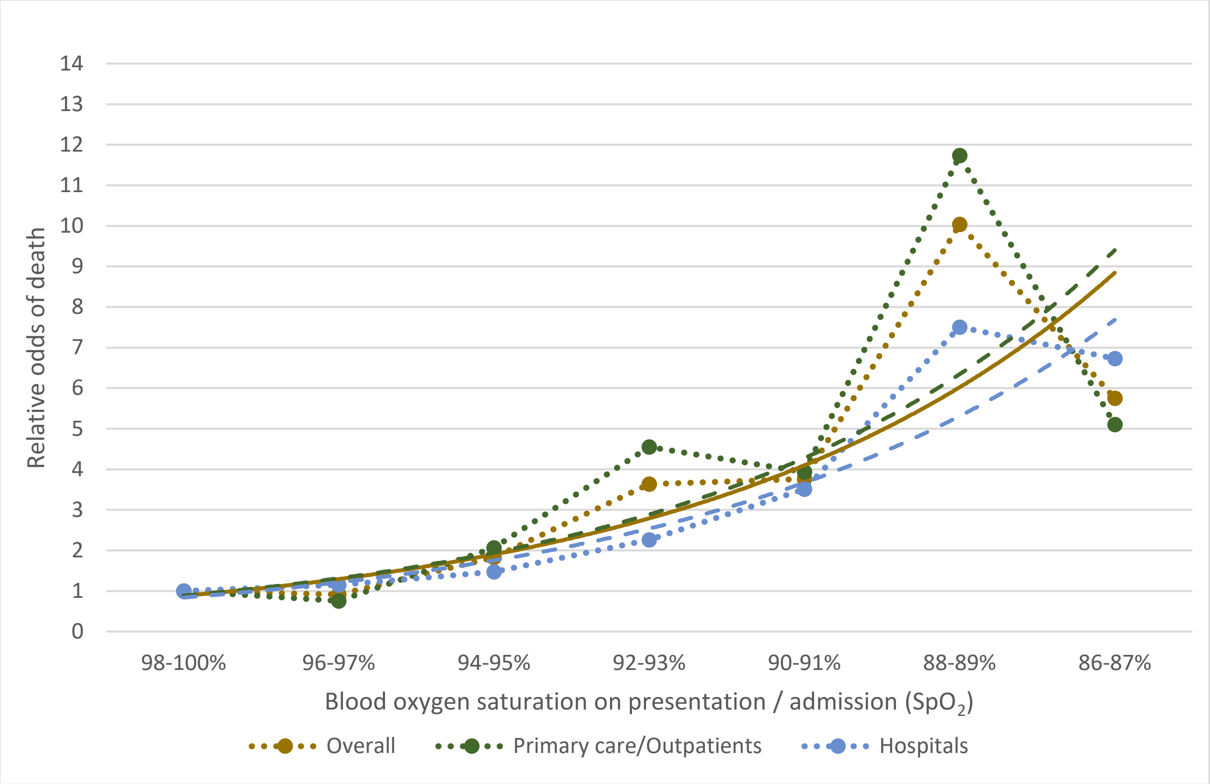


B) Original analysis extended to ≤85% group

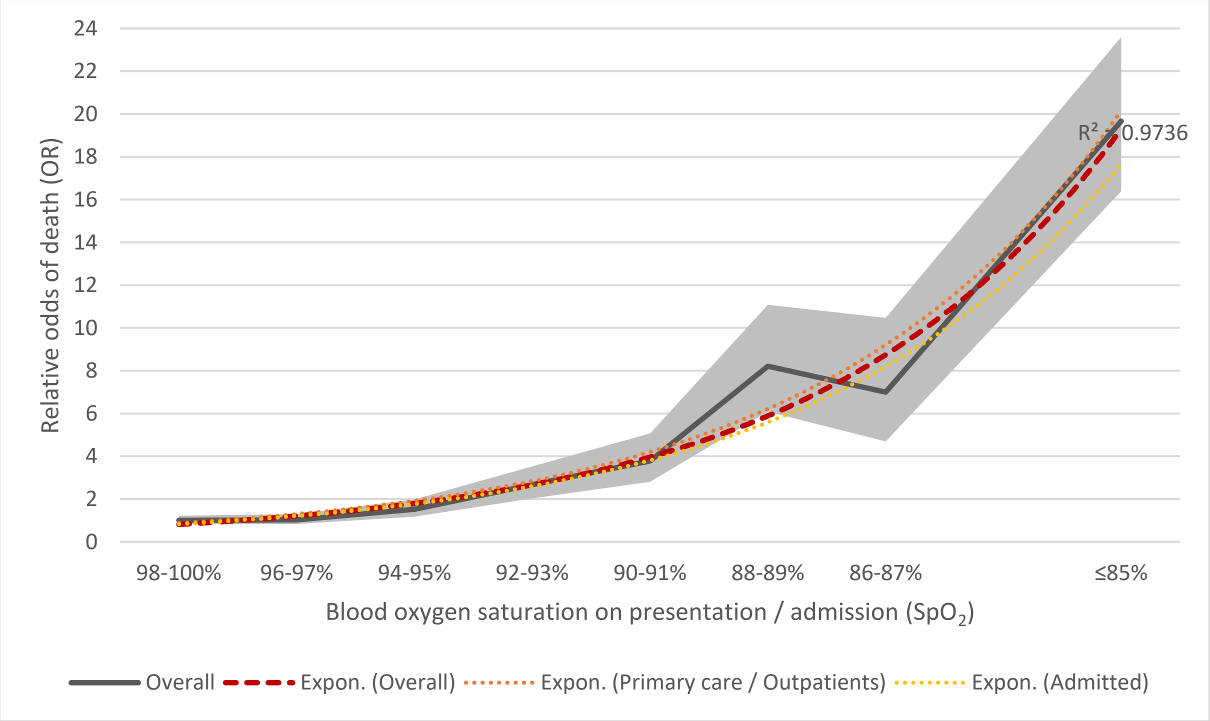

C) Original analysis showing Primary care / Outpatient total and 95% confidence interval

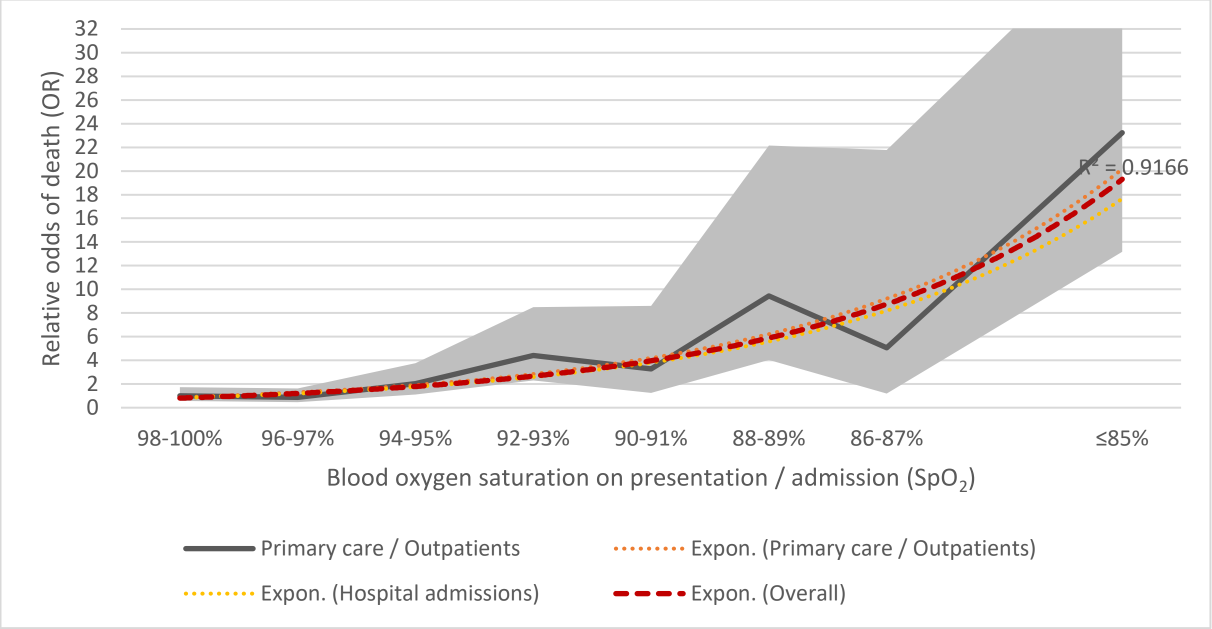

Supplement: Supplementary appendix [file mmc1.pdf]
